# γ-Secretase modulators exhibit selectivity for modulation of APP cleavage but inverse γ-secretase modulators do not

**DOI:** 10.1186/s13195-020-00622-5

**Published:** 2020-05-19

**Authors:** Christian B. Lessard, Edgardo Rodriguez, Thomas B. Ladd, Lisa M. Minter, Barbara A. Osborne, Lucio Miele, Todd E. Golde, Yong Ran

**Affiliations:** 1 Department of Neuroscience and Neurology, Center for Translational Research in Neurodegenerative Disease, and McKnight Brain Institute, College of Medicine, University of Florida, 1275 Center Drive, PO Box 100159, Gainesville, FL 32610 USA; 2 Department of Pharmacology and Therapeutics, Center for Translational Research in Neurodegenerative Disease, and McKnight Brain Institute, University of Florida, Gainesville, FL 32610 USA; 3 Department of Veterinary and Animal Sciences, Center for Bioactive Delivery, Institute for Applied Life Sciences, and Program in Molecular and Cellular Biology, University of Massachusetts, Amherst, MA 01003 USA; 4 Department of Genetics and Stanley S. Scott Cancer Center, Louisiana State University Health Sciences Center, New Orleans, LA 70112 USA

**Keywords:** Presenilin, Gene knockout, γ-Secretase modulators (GSMs), Amyloid peptide, Amyloid protein precursor (APP), NOTCH, VEGFR1, Alzheimer’s disease, Cancer

## Abstract

**Background:**

γ-Secretase is a multiprotein protease that cleaves amyloid protein precursor (APP) and other type I transmembrane proteins. It has two catalytic subunits, presenilins 1 and 2 (PS1 and 2). In our previous report, we observed subtle differences in PS1- and PS2-mediated cleavages of select substrates and slightly different potencies of PS1 versus PS2 inhibition for select γ-secretase inhibitors (GSIs) on various substrates. In this study, we investigated whether γ-secretase modulators (GSMs) and inverse γ-secretase modulators (iGSMs) modulate γ-secretase processivity using multiple different substrates. We next used HEK 293T cell lines in which *PSEN1* or *PSEN2* was selectively knocked out to investigate processivity and response to GSMs and iGSMs.

**Methods:**

For cell-free γ-secretase cleavage assay, recombinant substrates were incubated with CHAPSO-solubilized CHO or HEK 293T cell membrane with GSMs or iGSMs in suitable buffer. For cell-based assay, cDNA encoding substrates were transfected into HEK 293T cells. Cells were then treated with GSMs or iGSMs, and conditioned media were collected. Aβ and Aβ-like peptide production from cell-free and cell-based assay were measured by ELISA and mass spectrometry.

**Result:**

These studies demonstrated that GSMs are highly selective for effects on APP, whereas iGSMs have a more promiscuous effect on many substrates. Surprisingly, iGSMs actually appear to act as like GSIs on select substrates. The data with *PSEN1* or *PSEN2* knocked out HEK 293T reveal that PS1 has higher processivity and response to GSMs than PS2, but PS2 has higher response to iGSM.

**Conclusion:**

Collectively, these data indicate that GSMs are likely to have limited target-based toxicity. In addition, they show that iGSMs may act as substrate-selective GSIs providing a potential new route to identify leads for substrate-selective inhibitors of certain γ-secretase-mediated signaling events. With growing concerns that long-term β-secretase inhibitor is limited by target-based toxicities, such data supports continued development of GSMs as AD prophylactics.

## Background

γ-Secretase was originally identified as the protease that carried out the final cleavage to release the amyloid beta (Aβ) from the amyloid β precursor protein (APP) and was an early target for pharmacologic inhibition of Aβ by γ-secretase inhibitors (GSIs) in Alzheimer’s disease (AD) [1, 2]. γ-Secretase cleaves hundreds of type I transmembrane proteins, typically following ectodomain shedding, though it cleaves some full-length proteins as well [3]. γ-Secretase mediates signaling events by cleavage within the substrate’s transmembrane domain (TMD). This cleavage untethers the intracellular domain (ICD) from the membrane, allowing these domains to transduce signals by distributing to other sites within the cell [4]. In other cases, this cleavage can terminate a signaling event.

The role of γ-secretase in both APP and Notch1 biology has been extensively studied. Notch1 and APP both undergo sequential proteolysis. After ectodomain cleavage by a sheddase, the transmembrane carboxyl terminal fragments (CTFs) of Notch1 and APP are processed by γ-secretase in a stepwise manner [2]. γ-Secretase initially cleaves CTFs at a site near the cytoplasmic face of the membrane (S3 or ε-cleavage) and that cleavage is then followed by a 3 to 5 sequential di, tri, or tetra-peptide cleavages (S4 or γ -cleavage) [2, 4–7].

γ-Secretase cleavage eventually releases two potentially biologically active fragments: the intra cellular domain (ICD) and lumen or secreted small peptides. The signal transduction activity of NOTCH ICD (NICD) has been well validated, and signal transduction roles for APP ICD (AICD) and other intracellular domains released by γ-secretase have also been reported [3, 4]. Small peptides (Aβs) from APP have been extensively investigated due to their roles in AD; however, the secretion and function of NOTCH peptide (Nβs) or other small peptides putatively released by the combined action of the sheddase and γ-secretase largely remain unknown [8].

The sequential carboxyl peptidase-like cleavages of γ-secretase are referred to as γ-secretase processivity. For APP, processivity determines the length and amount of Aβ species released [9]. As longer Aβs aggregate into amyloid fibrils and other assemblies more readily than the shorter ones, regulation of γ-secretase processivity is a critical determinant of AD risk and remains a viable alternative to inhibition of γ-secretase or β-secretase as an AD therapeutic strategy. Notably, processive γ-secretase of APP can be altered either genetically or pharmacologically [1].

γ-Secretase is a multi-component protease including presenilin 1 and presenilin 2 (PS1 and PS2) forming the catalytic core and three accessory proteins (Nicastrin, APH1, and PEN-2) that regulate complex maturation, stability, and activity [10–13]. Mutations of PS1, PS2, and APP linked to familial forms of AD decrease γ-secretase processivity leading to increased relative levels of longer Aβ42 and sometimes Aβ43 peptides [9, 14]. These longer peptides aggregate more readily than shorter Aβ peptides and, in the absence of internal mutants in Aβ, appear to be required for deposition. Numerous small molecules, including some endogenous metabolites (e.g., cholestenoic acid), can modify γ-secretase processivity of APP. Such compounds are referred to as γ-secretase modulators (GSMs) [15–17]. Classic GSMs shift Aβ profile from longer species (Aβ43, 42 and 41) to shorter species (Aβ40, 38 and 37) by enhancing processivity. In contrast, a group of compounds called inverse GSMs (iGSMs) increase Aβ42 levels by decreasing processivity, in many cases with an accompanying decrease in shorter Aβ levels [18, 19].

Considerable efforts were devoted to developing GSIs in AD. Most GSIs show significant toxicity likely to inhibition of cleavage by of other substrates including Notch1. A phase 3 human AD trial was halted due to lack of efficacy and evidence for worsening of cognitive symptoms [20, 21]. GSIs have now been repurposed for various cancers and other disorders therapies largely because of their inhibition of Notch1 cleavage and signaling [22, 23]. We have previously reported that GSIs in cancer clinical trials are pharmacologically and functionally distinct and that selective inhibition of PS1 or PS2 by a given GSI does not explain their pharmacologically and functional differences [24, 25]. Recently, Habets et al. reported that the PS1 inhibitor MRK-560 effectively decreased mutant NOTCH1 processing in T cell acute lymphoblastic leukemia (T-ALL) cell lines, which were found to selectively express only PS1-containing γ-secretase complexes, suggesting that limiting side effects might be avoided by selective targeting of PS1 or PS2 [26]. This therapeutic window appears to be attributable to the differential expression of PS1 and PS2 in tissues and not a difference in the function of PS1- or PS2-containing γ-secretases per se.

The activity of GSMs and iGSMs on substrate cleavages other than APP and Notch1 has not been extensively evaluated [27]. Further, the potential utility of GSMs and iGSMs outside of AD has never been explored. In this study, we first investigated whether GSMs and iGSMs modulate γ-secretase processivity of γ-secretase using multiple different substrates. These studies demonstrated that GSMs are highly selective for effects on APP, whereas iGSMs have a more promiscuous effect on many substrates. Surprisingly, iGSMs actually appear to act as like GSIs on select substrates. We next used recently developed HEK 293T cell lines in which *PSEN1* or *PSEN2* were selectively knocked out to investigate processivity and response to GSMs and iGSMs; these data reveal that PS1 has higher processivity and response to GSMs than PS2, but PS2 has higher response to iGSM.

Collectively, these data indicate that GSMs are likely to have limited target-based toxicity. With growing concerns that long-term β-secretase inhibitor is limited by target-based toxicities, such data supports continued development of GSMs as AD prophylactics. In addition, they show that iGSMs may act as substrate-selective GSIs providing a potential new route to identify leads for substrate-selective inhibitors of certain γ-secretase-mediated signaling events.

## Methods

### Generation of recombinant substrates and cell-free γ-secretase cleavage assay

cDNAs encoding mouse Notch1, human NOTCH 1, NOTCH 2, NOTCH 3, NOTCH 4, CD44, and VEGFR1 γ-secretase substrates were generated by gene synthesis conducted by Genscript (Piscataway, NJ, USA). The general design of the constructs was similar to a recombinant substrate (APP C100) that has been used by our group and others to assay Aβ production in in vitro γ-secretase assays [24]. All constructs contain an NH_2_-terminal amyloid β peptide (Aβ) epitope tag followed by the juxtamembrane region (JMD) of the given substrate and a FLAG tag (DYKDDDDK) at the COOH-terminal. For clarity, these substrates are referred to as recombinant substrates (e.g., Notch1 is rNOTCH1_sub_)_._ Substrate cDNAs were cloned into pET21 (Novagen, Billerica, MA, USA) for expression in bacterial cells. Recombinant substrates were purified as described before [24].

CHAPSO-solubilized CHO and HEK 293T cell membrane were prepared as described in previous report [7]. Twenty-five micrograms per milliliter of each substrate was incubated with the membrane (100 μg/ml total protein) in sodium citrate buffer (150 mM, pH 6.5, Roche Complete protease inhibitor added) for 2 h at 37 °C. GSM1, Compound 2 (synthesized by A. Fauq at the Mayo Clinic Chemical Core), cholestenoic acid (CA) (Avanti Polar Lipids), fenofibrate (Sigma), (*Z-LL*)_2_ ketone (ZLL) (Calbiochem), and LY411575 (Sigma, St. Louis, MO) were used at desired concentration. The reaction was terminated by placing tubes on ice until immunoprecipitation.

### Cell-based γ-secretase cleavage assay

For the cell-based assay, HEK 293T wild type, cell lines that expressed only PS1 (*PSEN1*^*+/+*^, *PSEN2*^−/−^; referred to as PS1 lines), cell lines that expressed only PSEN2 (*PSEN1*^−/−^, *PSEN2*^+/+^; referred to as PS2), and cell lines that did not express either (*PSEN1*^−/−^, *PSEN2*^−/−^; referred to as DKO) [25] were cultured in DMEM media (Thermo-Fisher) supplemented with 10% fetal bovine serum (GE, Logan, UT, USA) and 1% penicillin/streptomycin (Life Technologies, Grand Island, NY, USA). Plasmids containing APP C100 cDNA were transfected into above cells using polyethylenimine (PEI). Cells and conditioned media were used for WB, IP, and ELISA.

### Immunoprecipitation and mass spectrometry

Immunoprecipitation and mass spectrometry (IP-MS) of Aβ and Aβ-like peptides in cell-free assay or conditioned media were performed as previously described [7, 15, 24, 28–30]. Briefly, the peptides were immunoprecipitated using anti Aβ Ab5 antibody bound to sheep anti-mouse IgG magnetic Dynabeads (Life Technologies) and eluted with 0.1% trifluoroacetic acid (TFA) in water. COOH-terminal fragments (CTFs) were immunoprecipitated with anti-FLAG M2 magnetic beads (Sigma). Eluted samples were mixed 2:1 with saturated α-cyano-4-hydroxycinnamic acid (CHCA) matrix (Sigma) in a mixture of acetonitrile (60%) and methanol (40%) and loaded onto a CHCA pretreated MSP 96 grounded steel target (Bruker, Billerica, MA). Spectra were collected and processed with a Bruker Microflex LRF-MALDI-TOF mass spectrometer using the flexControl and flexAnalysis software.

### ELISA and Western blotting

Sandwich ELISAs used for Aβ detection were performed as previously described [7, 31]. Briefly, Aβ and Aβ-like peptides in conditioned media were captured with Ab5 antibody and detected with horseradish peroxidase labeled mAb 4G8 (Biolegend). Synthetic Aβ1-40 was used as standard. All ELISAs were developed with TMB substrate (KPL, Gaithersburg, MD, USA). Bis-Tris precast gels (Biorad, Hercules, CA, USA) were used for all SDS-PAGE. Monoclonal anti-FLAG M2 antibody (Sigma) and Aβ1-16 antibody 6E10 (Covance) were used for Western blotting.

## Results

### GSM action is restricted to APP whereas iGSMs alter cleavage of other substrates

**Fig. 1:**
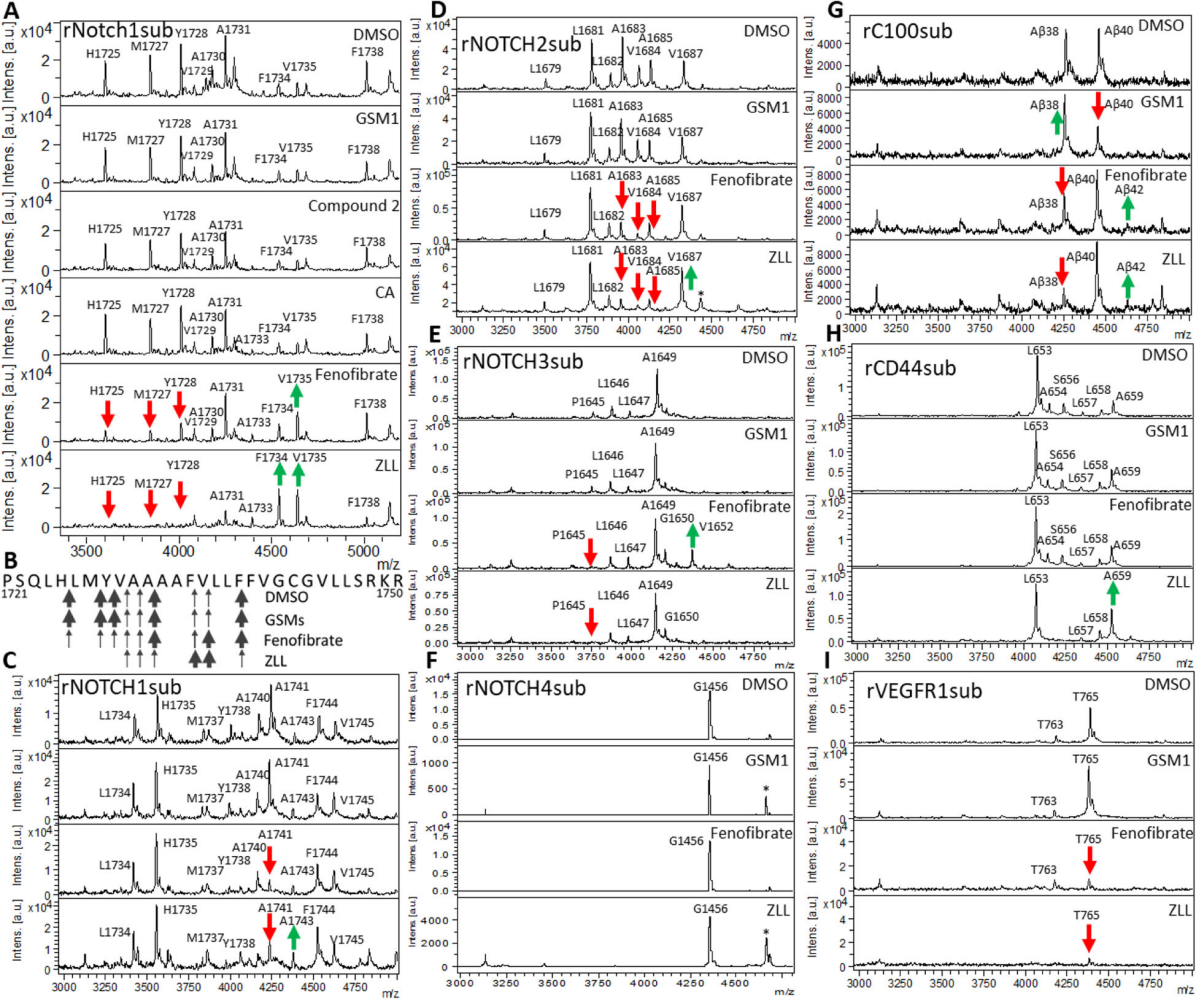
iGSM shifts γ-secretase process of many substrates beside rC100sub, but GSMs do not. Recombinant substrates were incubated with CHO cell membrane in the presence of GSM or iGSM. Aβ and Aβ-like peptide was immunoprecipitated with Ab5 antibody and analyzed by mass spectrometry (IP-MS). **a** rNotch1sub based on mouse Notch1 with DMSO, 1 μM GSM1, 1 μM Compound 2, 5 μM CA, 100 μM fenofibrate, or 12.5 μM ZLL. **b** Putative mouse Notch1 transmembrane domain and main cleavage sites observed in this study with GSMs and iGSMs. Weight of arrows indicates peak intensity in the spectra. **c**–**i** rC100sub, rNOTCH1-4sub, rCD44sub and rVEGFR1sub with DMSO, 1 μM GSM1, 100 μM fenofibrate, or 12.5 μM ZLL. * Unidentified peak (larger spectra are shown in Additional file 1)

GSMs have been shown to alter APP processing but not mouse Notch1; however, Wanngren et al. reported that a second generation GSM did modulate human Nβ [27]. We tested selected GSMs and iGSMs on a recombinant mouse Notch1 substrate (rNotch1sub) in an in vitro γ-secretase activity assay and used IP/MS to evaluate Nβ production. Nine Nβ peptides were observed in the spectra. All cleavage sites were located inside the putative transmembrane motif. None of the three GSMs used in this study, GSM1, Compound 2, and CA, altered the γ-secretase cleavage of rNotch1sub (Fig. 1a, b). In contrast, the two iGSM, fenofibrate and ZLL, dramatically altered the peptide profiles. The peak intensity of the three major short species (denoted by cleave sites within the precursor of H1725, M1727, and Y1728) decreased, and two larger species (V1735 and F1738) increased in the 100 μM fenofibrate-treated sample. The iGSM ZLL (12.5 μM) dramatically reduced shorter peptide cleavages (H1725 to A1731) and increased longer peptides (F1734 and V1735).

We next tested GSM1 and two iGSM, fenofibrate and ZLL, on panel of recombinant human substrates (NOTCH1-4, CD44, VEGFR1, and APP) using our in vitro assay (Fig. 1c–i). GSM1, fenofibrate, and ZLL shifted APP cleavage, as expected. However, GSM1 did not significantly change processivity of any of other substrates. In contrast, iGSMs had effects on cleavage of the other substrates. When tested using the rNOTCH1sub, fenofibrate selectively decreased the major cleavage product (A1741, corresponding to mouse rNotch1sub’s A1731) while other peaks remaining unchanged. ZLL also decreased A1741 and increased F1744. When tested using rNOTCH2sub, both fenofibrate and ZLL decreased three peptides (A1683, V1684, and A1685), and ZLL increased V1687. For the rNOTCH3sub, a minor peak (P1645) disappeared upon treating with fenofibrate and ZLL and a new cleavage peak at V1652 was detected with fenofibrate treatment. A single peak at G1456 was observed in rNOTCH4sub cleavage, and it was not changed by the iGSMs. For rCD44sub, neither GSM1 or fenofibrate treatment altered the cleavage profile, but ZLL increased A659 production comparing to the major cleavage at L653. In contrast to these substrates in the presence of fenofibrate or ZLL, γ-secretase cleavage of rVEGFR1sub was markedly inhibited. Extensive efforts to reproduce the cell-free IP-MS results in culture with PS1 and PS2 HEK cells were not successful.

### PS1 has higher processivity than PS2 in cell-based assay

**Fig. 2:**
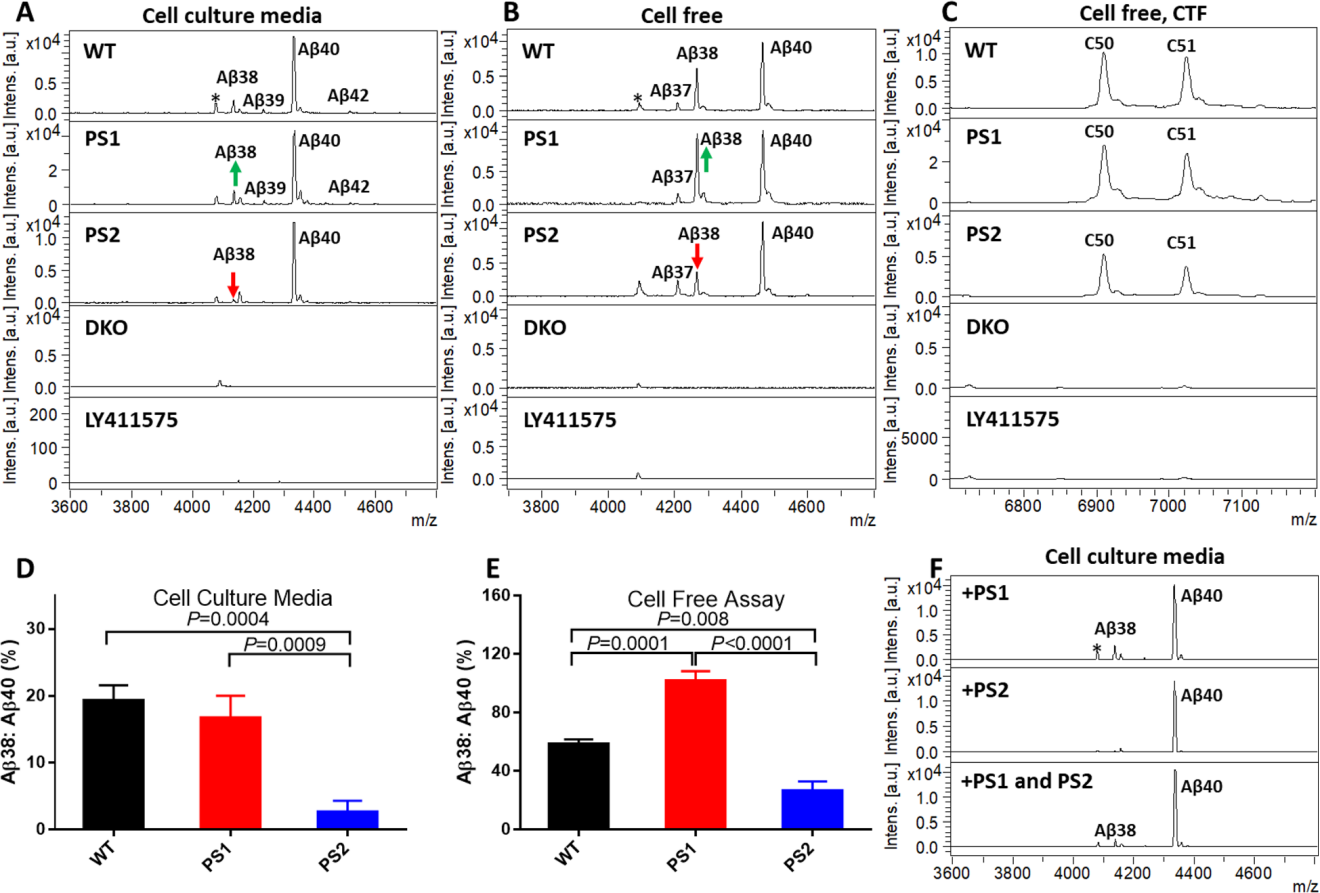
PS1 γ-secretase has higher processivity than PS2 γ-secretase on APP. **a** IP-MS of Aβ peptide in cell culture media. APPsw was transiently transfected into WT, PS1, PS2, and DKO HEK cells, and secreted Aβ peptides were immunoprecipitated with Ab5 antibody. **b** IP-MS of Aβ peptide in cell-free assay using WT, PS1, PS2, and DKO membranes with cC100sub as substrate. **c** IP-MS of CTF in cell-free assay. CTF was immunoprecipitated with anti-FLAG M2 antibody. **d**, **e** Summary results from mass spectrometry analysis presented in **a**, **b**. Histogram plots of the ratio of Aβ38 to Aβ40 in cell-based and cell-free assay (*n* = 3, one-way ANOVA, Tukey’s multiple comparisons test using Prism GraphPad version 8 software). **f** Transient transfection of PS1 and PS2 DNA into DKO cells shows similar processivity difference of PS1 and PS2. 0.5 μg PS1, PS2, or PS1+PS2 were co-transfected with 2 μg APPsw into DKO cells. IP-MS of Aβ peptide in conditioned media with Ab5 antibody. All experiments were repeated 3 times. Mass spectra from PS1-transfected cell show unidentified peaks (*)

Although we have previously reported that PS1 and PS2 are quite similar in terms of their substrate preferences [25], here, we more closely examined whether PS1 and PS2 generate different Aβ through altered processivity. For these studies, APPsw (APP Swedish) was transfected into WT and PS1 or PS2 only cells and the conditioned media were assayed with IP-MS. We found PS1 has higher processivity and generates relatively more Aβ38 than PS2 does (Fig. 2a). The ratio of Aβ38 to Aβ40 in WT, PS1, and PS2 cell media are 19.3%, 16.7%, and 2.6% respectively (Fig. 2d). Cell-free assay using WT, PS1, and PS2 cell membranes showed a similar pattern. The ratio of Aβ38 to Aβ40 was 58.4% (WT), 101.9% (PS1), and 26.5% (PS2), respectively (Fig. 2b, e). Notably, there were no detectable differences in the initial ε-cleavage. The ratio of the two major AICD products of 50 or 51 amino acids (C50 and C51), which are the counterparts of Aβ49 and Aβ48 respectively, did not change significantly. This difference in processivity was not attributable to clonal differences in the lines. Transient transfection of either PS1 or PS2 into our HEK 293T PS1/PS2 DKO cells showed comparable differences in production of Aβ38 (Fig. 2f).

### PS1 is more sensitive to GSMs whereas PS2 is more sensitive to iGSM

**Fig. 3:**
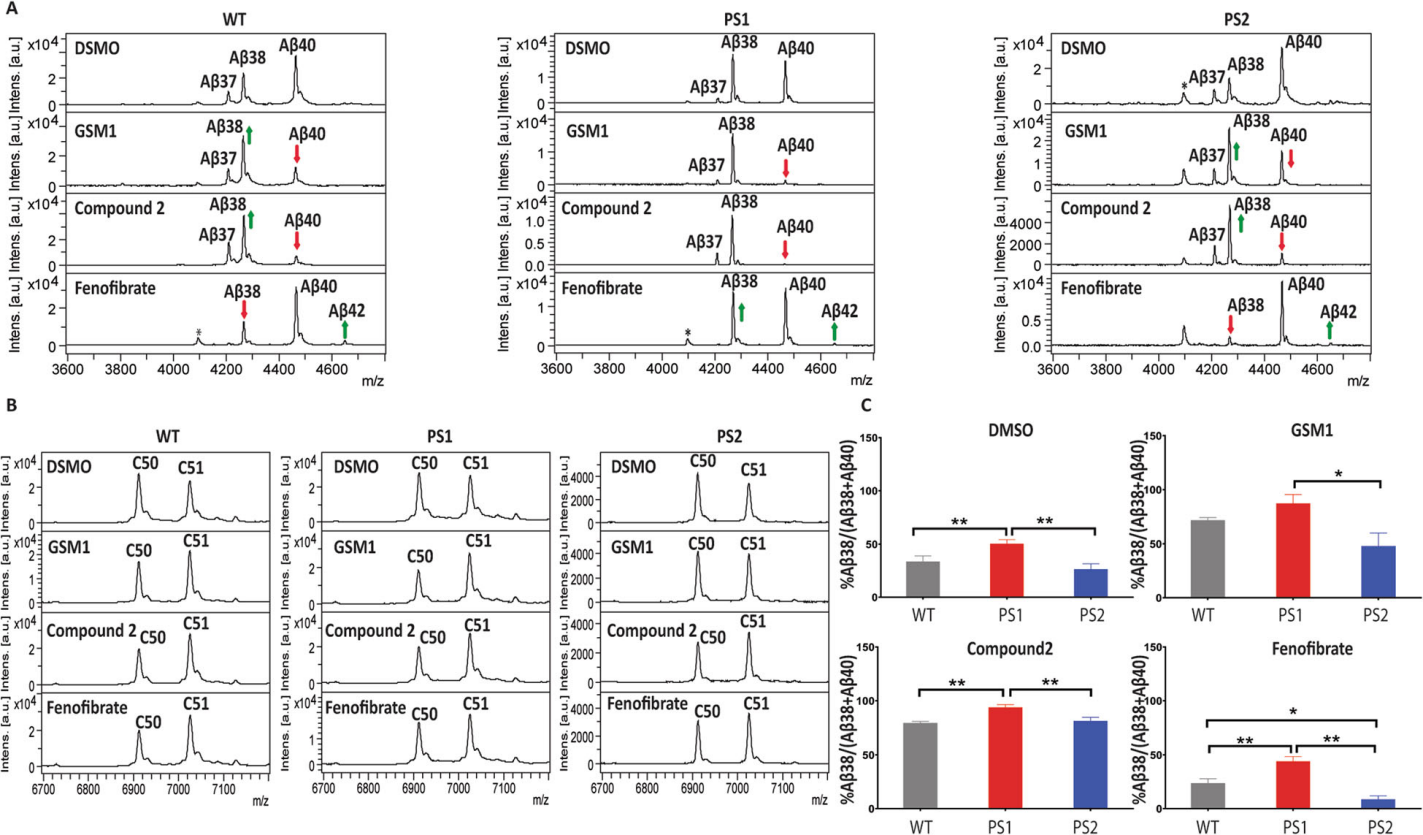
Cell-free assay shows GSM and iGSM affect PS1 and PS2 processivity with different efficiency. **a** 20 μg rC100sub was incubated with WT, PS1, or PS2 membrane in the presence of DMSO, 1 μM GSM1, 1 μM Compound 2, or 100 μM of fenofibrate. IP-MS of Aβ peptide with Ab5 antibody. **b** IP-MS of AICD with Anti-FLAG M2 antibody. **c** Percentage change of Aβ38 in cell-free assay with GSMs presented in **a**. The sum of peak intensities of Aβ38 and Aβ40 was set as 100%. Analysis of statistical significance was performed by one-way ANOVA with Tukey’s multiple comparisons test using Prism GraphPad version 8 software (*n* = 3, *p < 0.05, ** *p* < 0.01)

Several models have been proposed for how GSMs and iGSMs alter γ-secretase processivity. However, whether modulatory activity is different in PS1- or PS2-containing γ-secretase complexes has not been explored. Here, we tested the GSMs (GSM1 and Compound 2) and the iGSM (fenofibrate) on WT, PS1, and PS2 HEK membranes using our cell-free activity assay (Fig. 3a). GSM and iGSM treatment of the WT cell membranes showed the expected effects on Aβ peptide production. GSMs increased shorter Aβ peptides, and iGSMs had the opposite effect. In PS1 membranes, GSMs showed an enhanced effect toward increased processivity, best observed by the larger reduction in Aβ40 and larger increases in Aβ38. In contrast, the iGSM treatment resulted in only slight changes in the peptides produced. In PS2 membranes, GSMs showed less alterations in the Aβ profiles and iGSMs had an enhanced response when compared to WT. These shifts in response to GSM and iGSM treatment are shown quantitatively in Fig. 3c, where average peak heights of the spectra are depicted graphically. Notably, the increased effects of GSMs on PS1 can be observed by an increased effect on the amyloid intracellular domain (AICD) fragments generated, where there is a larger shift from C50 to C51 cleavage (Fig. 3b). Although not as definitive, GSMs appeared to have less effect on the AICD production in PS2 membranes and it appears that iGSMs had a larger effect.

## Discussion

We have extended previous studies showing that GSM activity is quite selective for APP with minimal effects on other substrates. In contrast, we find that iGSM activity is more promiscuous and that iGSMs do not show substrate selectivity. Further, under select circumstances, we find that iGSMs can inhibit rather than modulate γ-secretase cleavage of a few substrates. This later finding is unexpected but does suggest that it may be possible to find highly substrate-selective γ-secretase inhibitors derived from iGSMs. Future efforts focusing on increasing the potency of current iGSMs that act as substrate-selective GSIs may renew interest in generating highly potent and substrate-selective GSIs. Given the interest in the role of γ-secretase in mediating signaling events in cancer, immune function, and other non-AD research areas, it is likely that this finding may have broad relevance to those with interests in more selective modulation of γ-secretase activity. However, it is likely that increases in potency from the current micromolar range will be needed to advance such inhibitors for various clinical indications. No matter what given the pleiotropic effects γ-secretase on various signaling pathways and toxicity of pan γ-secretase inhibition, such substrate-selective inhibitors would be valuable tools for the field [1].

We also observed that PS1 and PS2 γ-secretases have subtle but significant different degrees of processivity and different sensitivities to GSMs and iGSMs, at least under the conditions tested. PS1 γ-secretase appears to be more processive, at least with respect to APP cleavage, is more sensitive to GSMs, and is more resistant to iGSMs. Conversely, PS2 γ-secretase is less processive, less sensitive to GSMs, and more sensitive to iGSMs. These data would suggest that the main action of GSMs would likely be on PS1 γ-secretase complexes and iGSMs is on PS2 γ-secretase complexes. The sensitivity of PS1 toward GSM-1 is consistent with another group who reported a higher binding affinity of GSM-1 on PS1-NTF over PS2-NTF [32]. However, the binding affinity of fenofibrate for PS1-NTF and PS2-NTF remained indicating another mechanism. It has been reported that iGMS tends to destabilize the γ-secretase complexes and the restricted cellular localization of PS2 could explain the higher avidity of iGMSs on PS2 [33, 34]. In any case, these data show that human PS1 and PS2 complexes have important differences in activity than can be revealed by pharmacological manipulations.

Much of the data in this report was generated using cell-free γ-secretase assays with recombinant substrates, with critical findings validated in cell lines, including the human PS1 and PS2 only HEK cell lines. Therefore, as has been suggested, a number of aspects of γ-secretase activity are not fully reconstituted in such in vitro systems [35]. For the most part, tools to readily validate these observations in more relevant models and in vivo are simply lacking.

## Conclusions

Therapies targeting Aβ production, clearance, or toxicity have yet to show appreciable clinical benefit, though recent clinical data from aggregate selective antibodies (aducanumab, BAN2401) shows hints of limited efficacy in symptomatic AD. As discussed in multiple reviews and perspectives, there are many reasons for these failures ranging from poor target engagement to treatment at the wrong stage of disease [36–38]. Indeed, both conceptually and in preclinical studies, there is little evidence that a GSM would have benefit in AD unless used prophylactically either in primary or possibly in secondary prevention paradigms.

Therapeutics that target Aβ and intended for use in primary or secondary prevention must have a high degree of safety. Given the recent reports showing that all of the clinical β-secretase inhibitors tested sufficiently show cognitive and neurological side effects, even in the preclinical stages of AD, there is a pressing need to find alternative approaches to targeting Aβ production that are “safe-enough” [39]. If we do not have such agents, we may never test the fundamental tenant of the amyloid cascade hypothesis that preventing amyloid deposition prevents AD [40]. The data here reinforce that GSMs show a high degree of specificity for APP. Further, the subtle functional effects of these compounds on γ-secretase processivity and not overall cleavage reduce concerns that GSMs would have on target toxicity. It has been challenging to develop GSMs with appropriate pharmacologic properties and sufficiently limited off-target toxicities. However, given the unexpected challenges with β-secretase inhibition, it may be wise for the field to renew efforts to find highly potent selective and safe GSMs with optimal pharmacological properties.

## Supplementary information

**Additional file 1.**

## Data Availability

The data of the current study are available from the corresponding author on reasonable request.

## Abbreviations

AICD: Amyloid intracellular domain
CTF: C-terminal fragment of APP
GSI: γ-Secretase inhibitor
GSM: γ-Secretase modulator
iGSM: Inverse γ-secretase modulator
IP-MS: Immunoprecipitation-mass spectrometry
PS1: Presenilin 1
PS2: Presenilin 2
