# Supplementary figures and images for: γ-Secretase modulators exhibit selectivity for modulation of APP cleavage but inverse γ-secretase modulators do not
Source: Alzheimers Res Ther. 2020 May 19;12:61. doi: 10.1186/s13195-020-00622-5 (PMC7236921; doi:10.1186/s13195-020-00622-5)

Larger Spectra of Figure 1. on multiple pages.

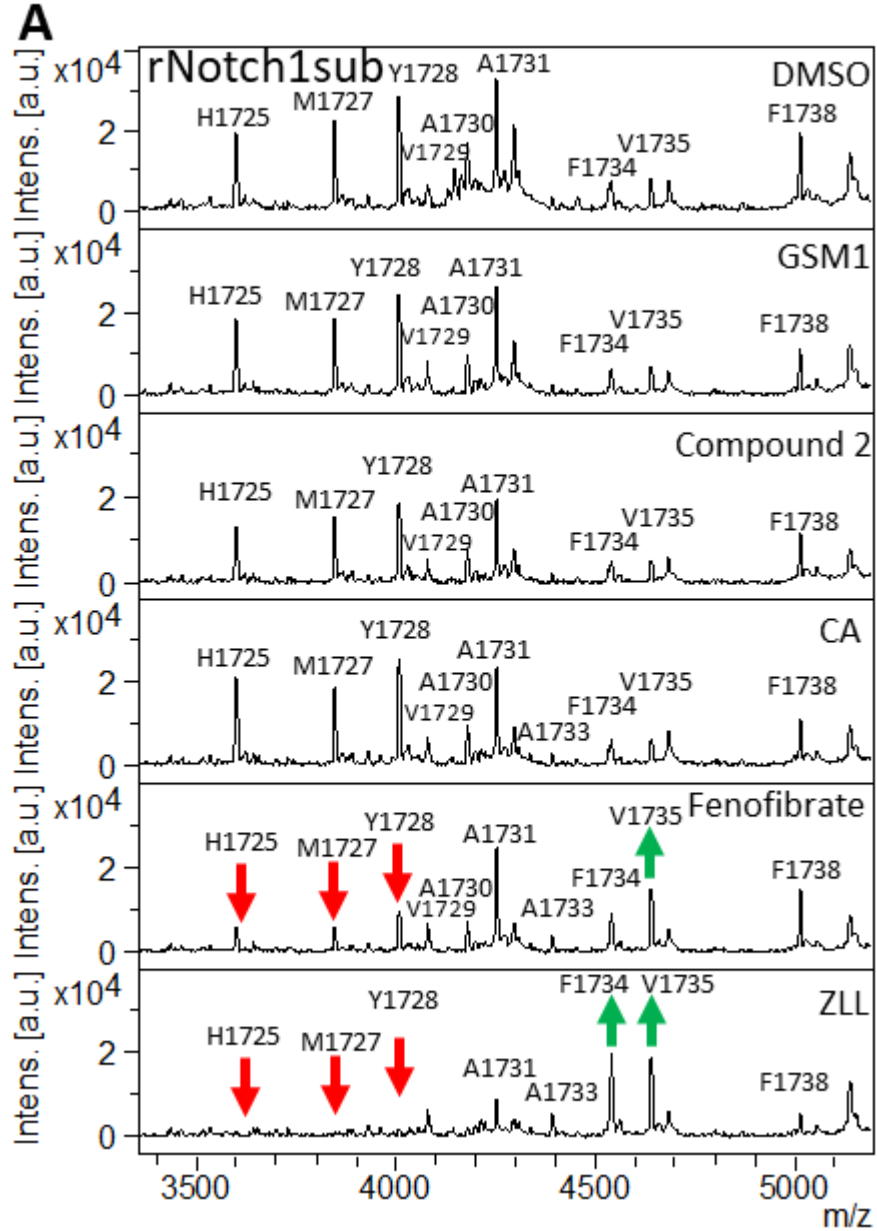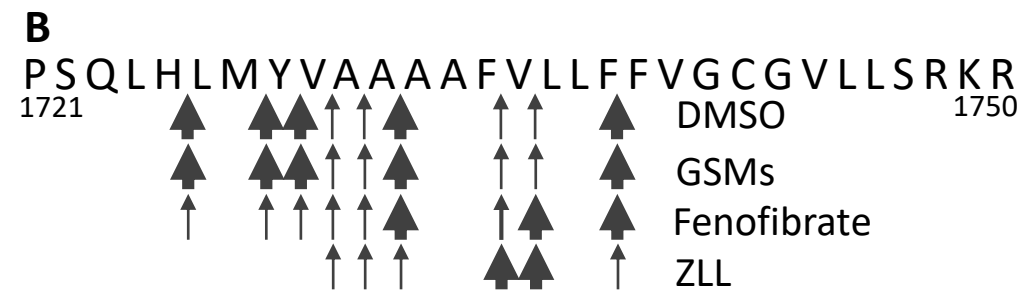

**C**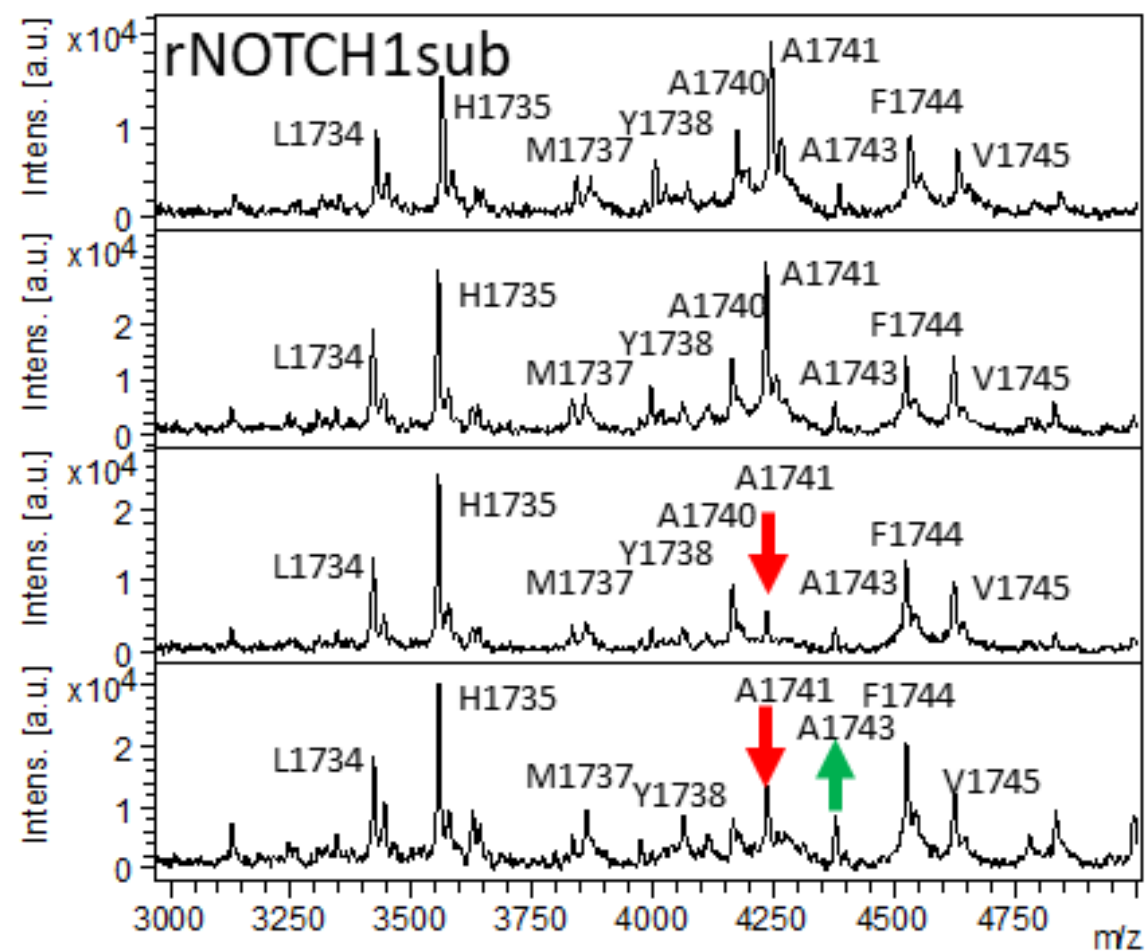**D**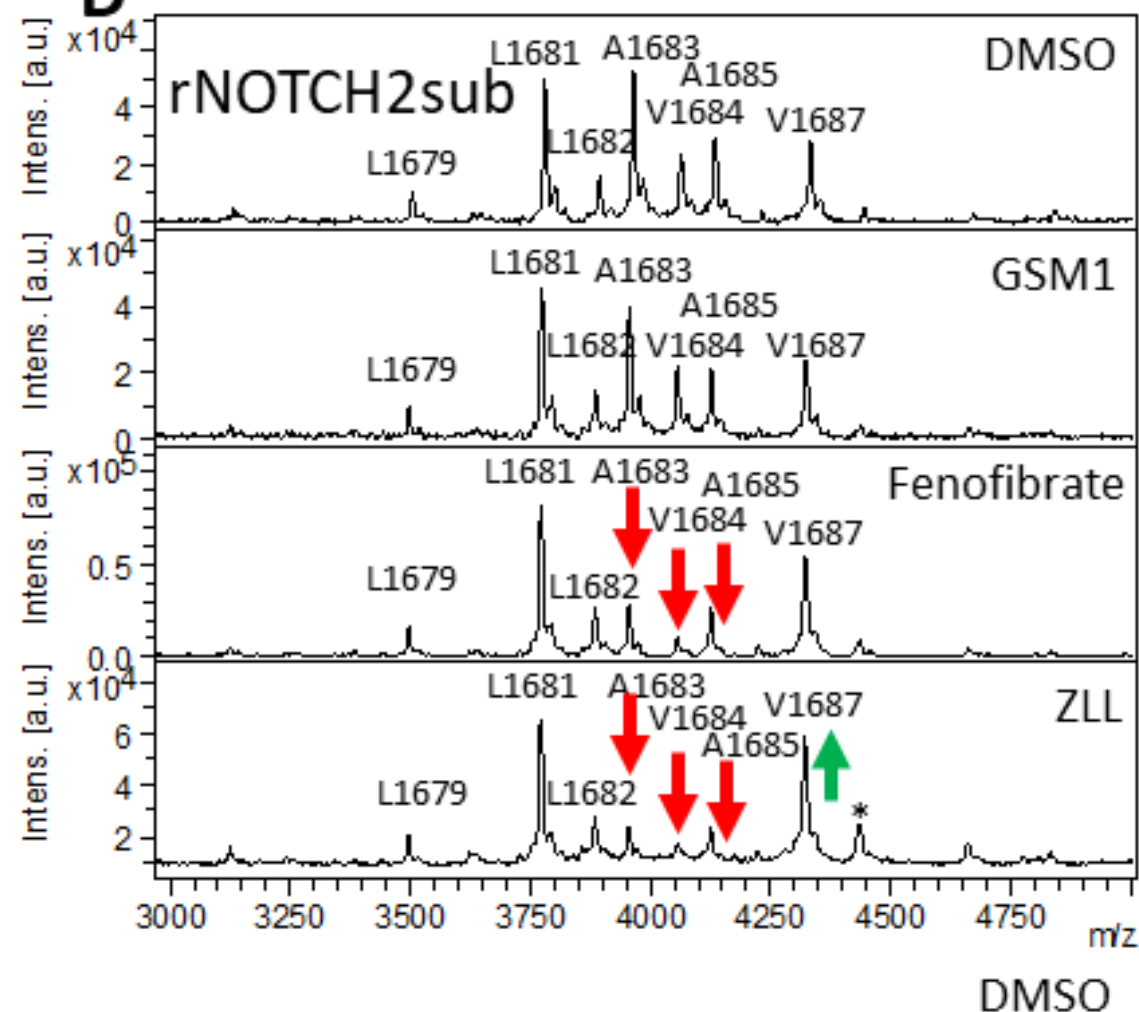

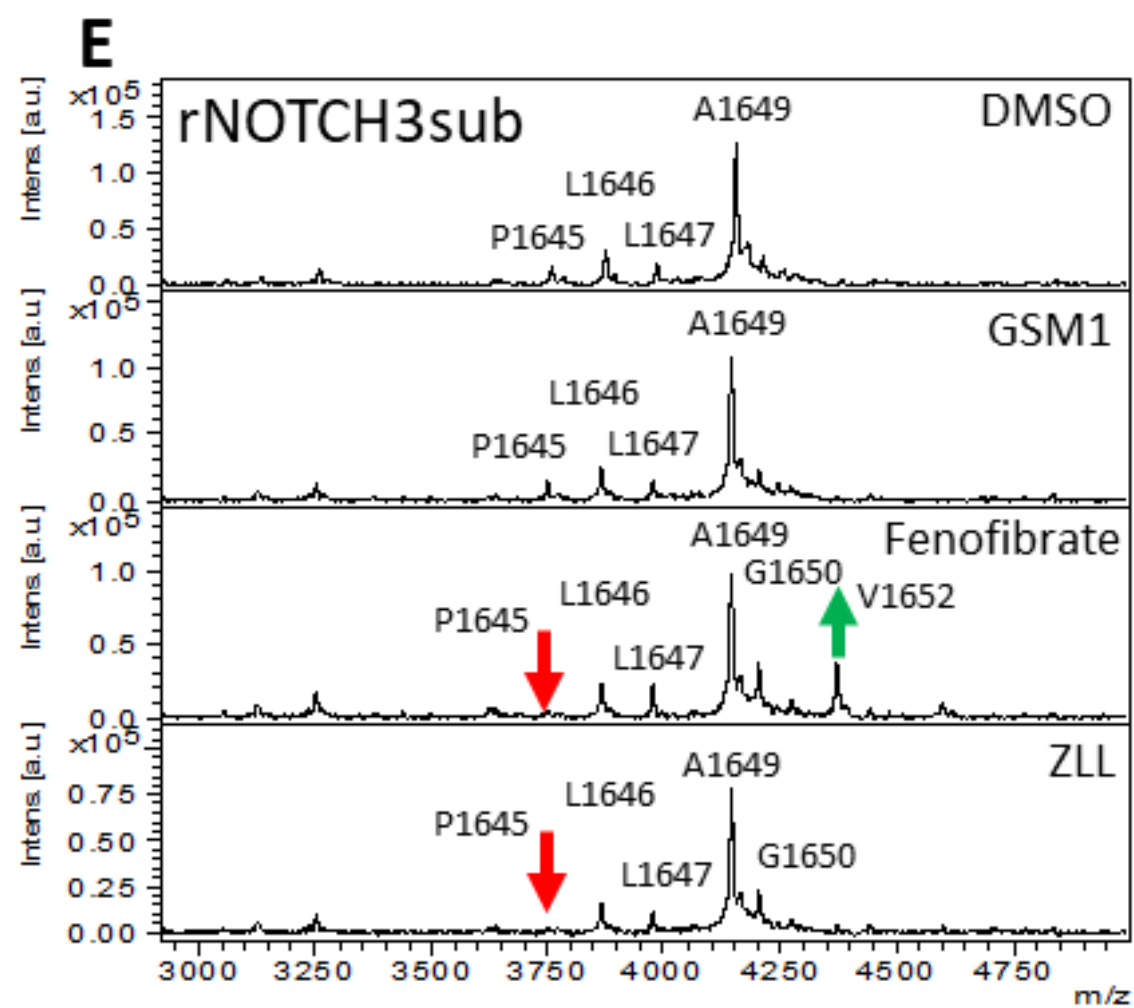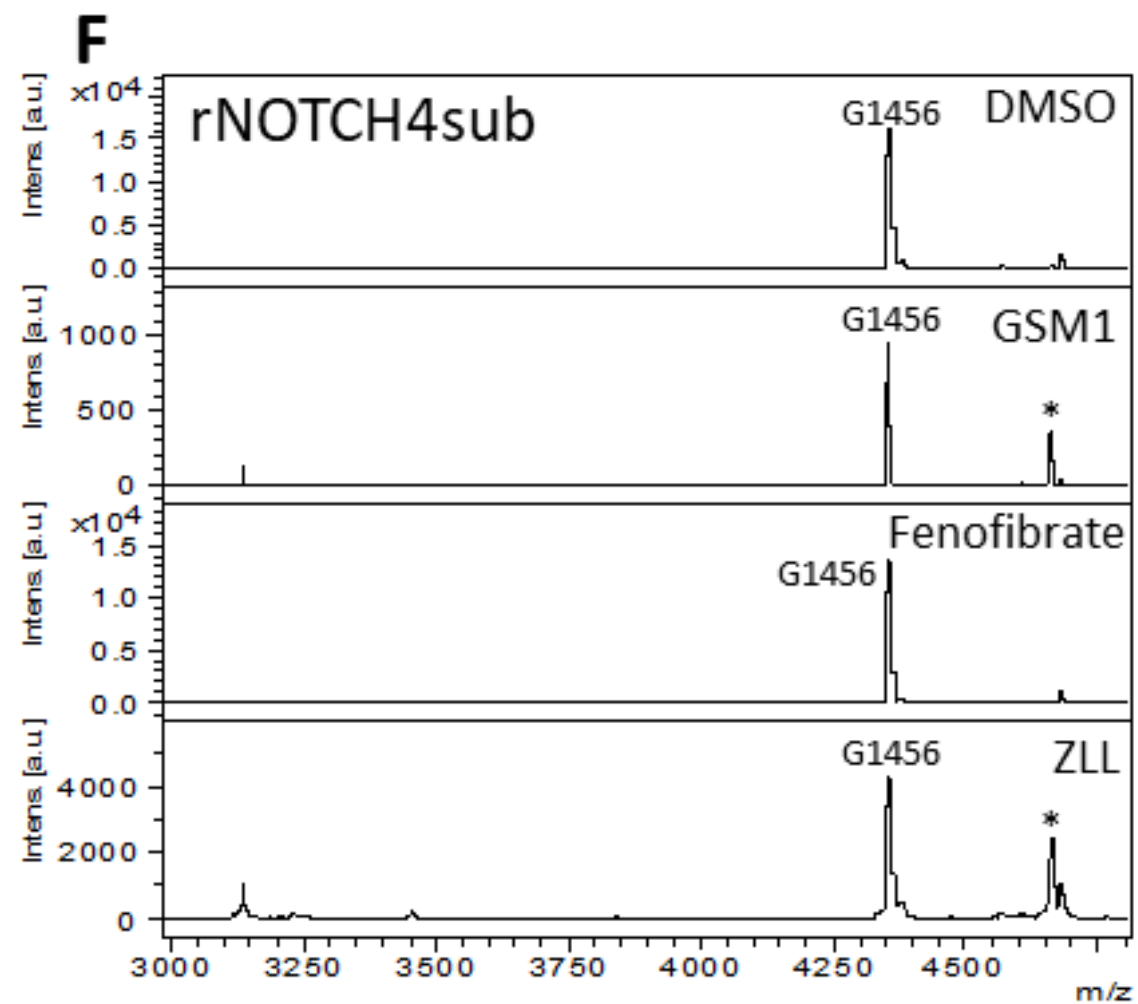

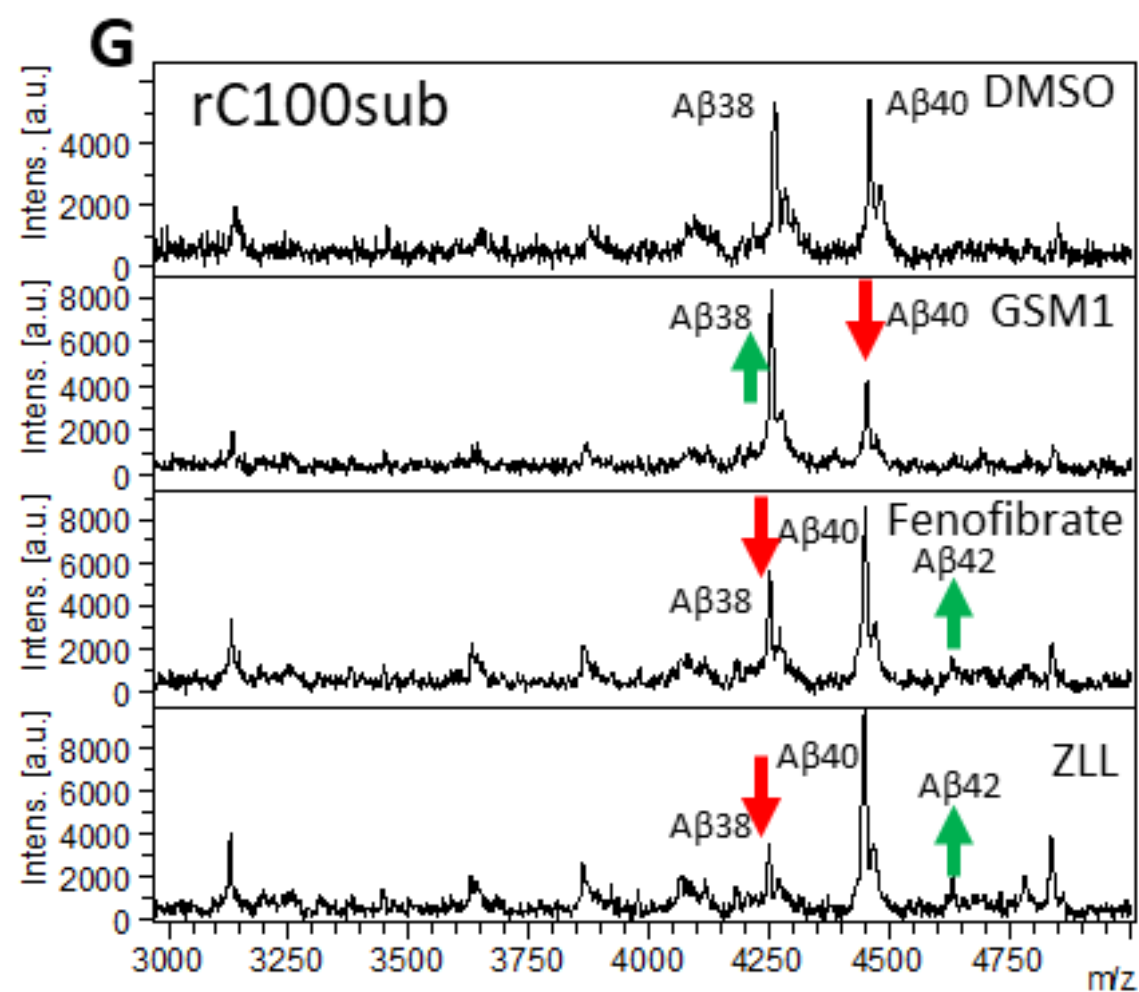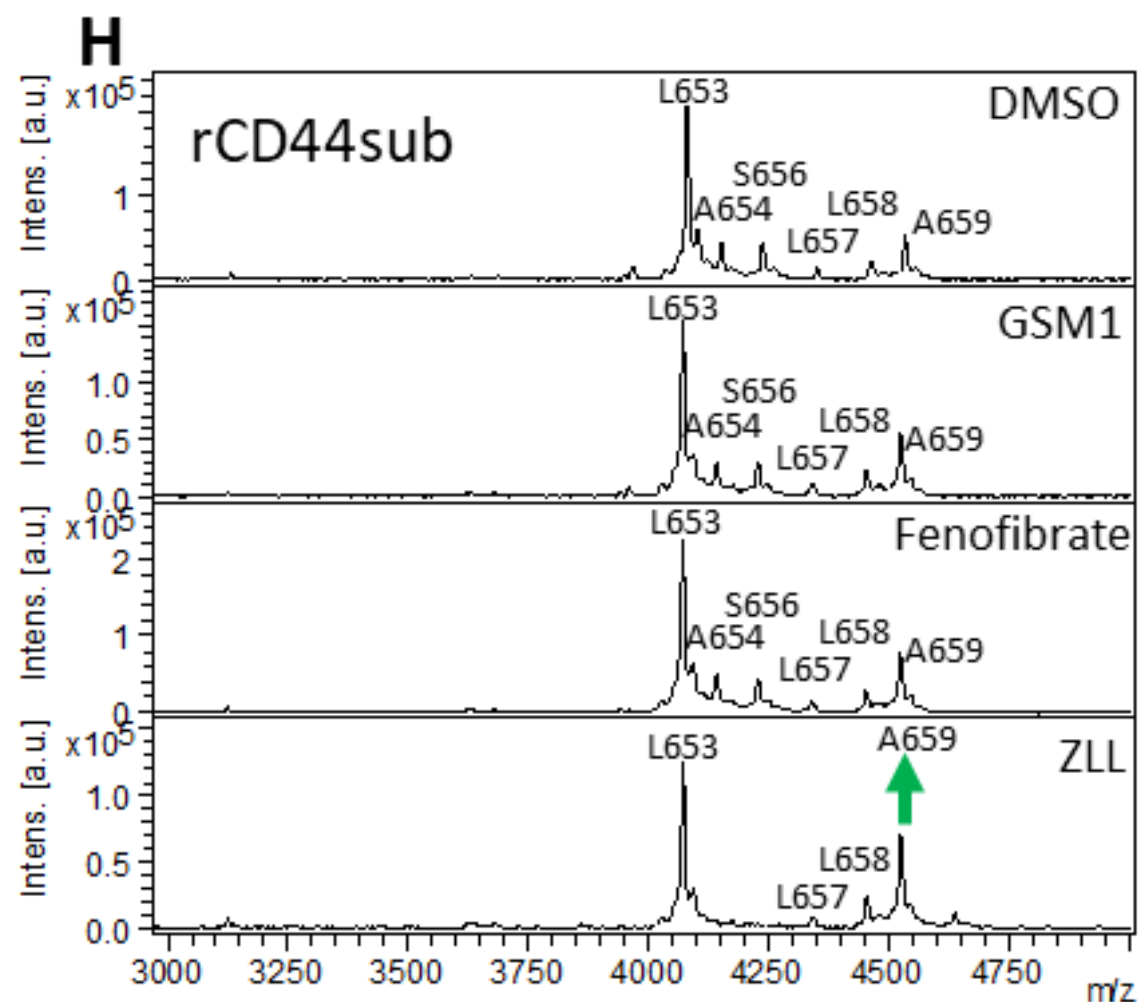

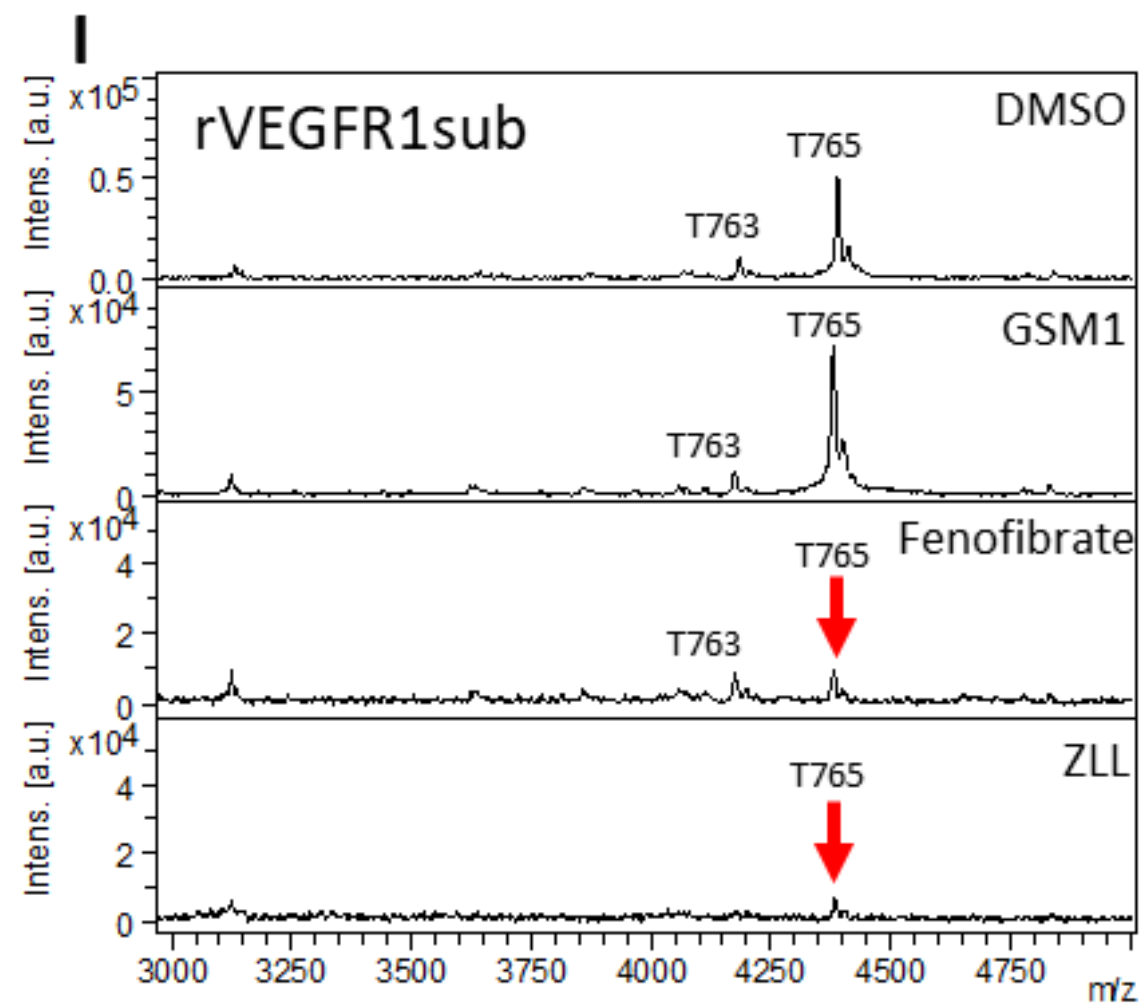

Supplement: Supplementary file 1 — Additional file 1. [file 13195_2020_622_MOESM1_ESM.pdf]
